# Electrophysiological Differences in the Processing of Affect Misattribution

**DOI:** 10.1371/journal.pone.0049132

**Published:** 2012-11-07

**Authors:** Yohei Hashimoto, Tetsuto Minami, Shigeki Nakauchi

**Affiliations:** 1 Department of Computer Science and Engineering, Toyohashi University of Technology, Toyohashi, Aichi, Japan; 2 The Electronics-Inspired Interdisciplinary Research Institute, Toyohashi University of Technology, Toyohashi, Aichi, Japan; University of Bern, Switzerland

## Abstract

The affect misattribution procedure (AMP) was proposed as a technique to measure an implicit attitude to a prime image [1]. In the AMP, neutral symbols (e.g., a Chinese pictograph, called the target) are presented, following an emotional stimulus (known as the prime). Participants often misattribute the positive or negative affect of the priming images to the targets in spite of receiving an instruction to ignore the primes. The AMP effect has been investigated using behavioral measures; however, it is difficult to identify when the AMP effect occurs in emotional processing—whether the effect may occur in the earlier attention allocation stage or in the later evaluation stage. In this study, we examined the neural correlates of affect misattribution, using event-related potential (ERP) dividing the participants into two groups based on their tendency toward affect misattribution. The ERP results showed that the amplitude of P2 was larger for the prime at the parietal location in participants showing a low tendency to misattribution than for those showing a high tendency, while the effect of judging neutral targets amiss according to the primes was reflected in the late processing of targets (LPP). In addition, the topographic pattern analysis revealed that EPN-like component to targets was correlated with the difference of AMP tendency as well as P2 to primes and LPP to targets. Taken together, the mechanism of the affective misattribution was closely related to the attention allocation processing. Our findings provide neural evidence that evaluations of neutral targets are misattributed to emotional primes.

## Introduction

Emotional stimuli automatically guide attention. Automatic processes play an important role in instantaneous emotional processing, which influences the subsequent reflective processing of affect [2]. For instance, previous studies [3]–[5] have demonstrated that the time to evaluate a target item as either “happy” or “sad” is shorter when the prime and target pairs are affectively congruent (e.g., positive-positive) than when they are affectively incongruent (e.g., positive-negative). This phenomenon is referred to as an affective priming effect. In addition, the emotional prime influences not only the evaluation time of the target, such as the affective priming effect, but also the emotional evaluation of the target itself. This phenomenon is referred to as an affect misattribution [1], [6]. The affect misattribution procedure (AMP) has been proposed as a technique to measure an implicit attitude toward a prime image [1]. Neutral symbols (e.g., a Chinese pictograph, called the target) are presented, following an emotional stimulus (the prime). Participants are instructed to ignore the prime picture and to rate the pleasantness (i.e., whether like or dislike) of the target pictographs. During these trials, they often misattribute the positive or negative affect of the priming images to the targets in spite of the instruction to ignore the primes (e.g., a positive response to a neutral target that follows positive primes).

The AMP has become an important task in the field of implicit social cognition [7]. The AMP effect has been investigated using behavioral performance measures [6], [8], [9] and has been shown to be influenced by the interval between the onset of the prime and the onset of the target, which is referred to as stimulus onset asynchrony (SOA). The effect was significantly decreased with a long SOA, which suggested that it is possible to correct for the influence of the primes with slow time scales [1]. Oikawa and colleagues addressed the mechanism of affect misattribution by examining the consequences of the explicit evaluation of primes [6]. The affect misattribution effect was observed when participants ignored the primes; however, this effect disappeared when participants had to rate the prime before rating the pictograph. The researchers reasoned that the affect became bound to the prime and therefore could not be misattributed to the pictograph anymore.

The behavioral approach has provided important information concerning the AMP effect; however, it is difficult to identify when the AMP effect occurs in emotional processing—whether the effect may occur in the earlier attention allocation stage or in the later evaluation stage. Event-related brain potentials (ERP) with a high temporal resolution have facilitated studies with the purpose of identifying the temporal stages of the AMP effect. Previous ERP studies have shown that the late positive potential (LPP, beginning around 500 ms post stimulus) and the earlier visual P2 component (beginning around 200 ms post stimulus) are both strongly sensitive to the emotional valence of a stimulus, regardless of whether evaluations are implicit or explicit [10]–[13]. Some studies suggest that early components may be able to index relatively automatic increases in selective attention, whereas late components may be associated with the evaluative processes following the presentation of emotional stimuli [11], [14], [15].

On the other hand, the neural correlates of affective priming [16], whose method is similar to AMP, have been studied. Both methods measure the affective effect of primes on targets. Zhang et al. (2010) studied the ERP correlates of cross-domain affective priming using picture-word pairs in which participants were asked to decide whether the valence of each target word following the prime picture was pleasant or unpleasant [5]. They found that incongruent pairs evoked a larger LPP than congruent pairs across the scalp at an SOA of 250 ms. Werheid and colleagues (2005) used emotional face pairs to examine the ERP correlates of affective priming in an evaluative decision task in which participants were asked to classify portraits of unfamiliar persons according to their emotional expression (happy or angry) [17]. They suggested that at an SOA of 250 ms, ERP results revealed both early and late priming effects, independent of the stimulus valence.

As mentioned above, the correlation of the priming effect of emotional stimuli and ERP has been investigated. As for affective priming and AMP, both tasks are similar on the point of measuring the affective effect of primes on targets. However they are substantially differs in methodological details and in the task-specific mechanism underlying the measure [18]. The different point is that AMP influences the evaluation itself, whereas affective priming influences its reaction times based on response interference. Therefore, the neural correlates of affective priming and AMP are thought to be different, and it is still unclear how AMP modulates early and late processing. In this study, we investigate the temporal dynamics of the AMP process using EEG. Through an ERP analysis, we investigated early processing for the prime in addition to early processing and late processing for the target. We supposed that a difference in neural processing for the prime could explain the tendency toward affect misattribution; hence, we classified participants by their tendencies toward affect misattribution and investigated how the tendencies of affect misattribution and the ERP components were correlated.

## Methods

### Participants

Twenty volunteers (16 men and 4 women) participated in the study. The mean age of participants was 24.6 years (ranging from 22 to 39). An informed written consent was obtained from participants after details of the procedure had been explained to them. The experimental procedures were approved by the Committee for Human Research of Toyohashi University of Technology.

### Stimuli

The pictures used as primes consisted of 80 negative, 80 neutral, and 80 positive pictures from the International Affective Picture System (IAPS) [19]. The mean valence on a 1–9 point scale (with 9 being the most positive) was 2.89 (SD = 0.37) for negative pictures, 5.07 (SD = 0.61) for neutral pictures, and 7.44 (SD = 0.33) for positive pictures, respectively. The mean arousal on a 1–9 point scale (with 9 being the highest arousal) was 4.96 (SD = 0.46) for negative pictures, 4.96 (SD = 0.75) for neutral pictures, and 4.98 (SD = 0.49) for positive pictures, respectively. The pictures with large gender difference (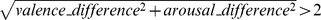 were excluded from the stimuli. Yi pictographs, which are used in some parts of China, were used as targets. 80 neutral targets were selected from 336 Yi pictographs by conducting the pilot study (n = 4). The participants' task was to judge the pleasantness (in terms of like or dislike) of the Yi pictographs. We selected the Yi pictographs whose proportion of “pleasant” responses was 50% as target stimuli (the mean proportion was 54.2 (SD = 22.2)).

### Procedure

The experiments were performed in a dark room, and stimuli were displayed on a TOTOKU CV921X CRT monitor with a spatial resolution of 800×600 pixels and refresh rate of 100 Hz, driven by a VSG2/5 graphics card (Cambridge Research Systems). The participants were seated in front of a computer screen at a distance of 55 cm, and all stimuli were presented with a visual angle of 6×8° on a medium-gray background.

**Figure 1:**
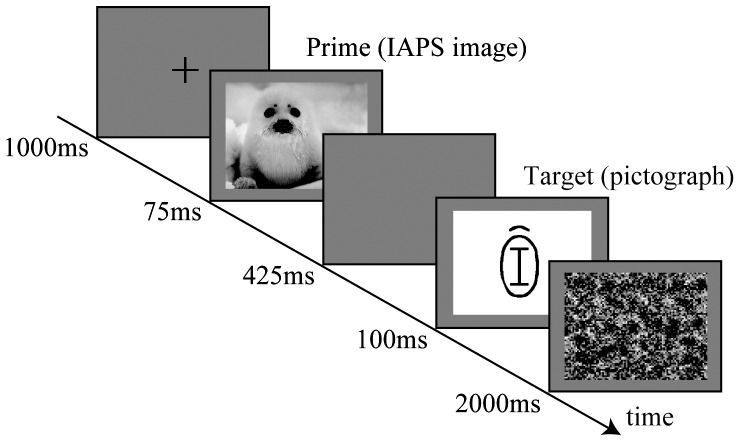
Stimuli and experimental procedure. In each trial, a prime picture (IAPS) was presented for 75 ms following a fixation point for 1000 ms. After a blank screen was shown for 425 ms, a target pictograph was presented for 100 ms and a noise for 2000 ms. This study involved 3 blocks. Each block comprised 80 trials. Each prime picture was presented only once, and each target pictograph appeared 3 times.

We adapted the AMP based on a previous study [1]. Figure 1 illustrates the experimental procedure. In each trial, a prime picture (IAPS) was presented for 75 ms following a fixation point for 1000 ms. After a blank screen was shown for 425 ms, a target pictograph was presented for 100 ms, and a noise for 2000 ms. This study involved 3 blocks, each block comprising 80 trials. Each prime picture was presented only once, and each target pictograph appeared three times (each target appeared once after each prime). Stimuli were presented in a pseudo-randomized order.

The participants' task was to judge the pleasantness (in terms of like or dislike) of the target pictograph and to take no account of the prime picture. Participants were told that the photographs presented prior to the target pictographs could bias their responses on those pictographs and that they should try to make sure that their responses were not influenced by the photographs. Participants were asked to press one of two buttons to indicate their choices. Trials with a longer response time (>2000 ms from target presentation) were removed from the analysis. The assignment of the response hand was counterbalanced across the participants.

To confirm a misattribution effect for prime pleasantness, the proportions of “pleasant” responses to the target pictograph (“like” response) in each prime condition were calculated for each individual participant. Because we wished to examine whether the effect of the intensity of misattribution was associated with a neural response, the misattribution index (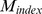) was calculated for each individual participant.
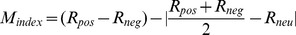


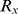
 indicates the proportion of “pleasant” responses to the prime with valence:x (pos: positive, neg: negative, and neu: neutral valence). Participants were separated into the two groups based on the 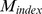; participants with 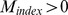 were allocated a low tendency to misattribution, and participants with 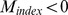 were allocated a high tendency. A 3 prime valence (negative, neutral, and positive)×2 misattribution tendency (high, low) repeated measures ANOVA was performed for proportions of “pleasant” responses.

### EEG recording and analysis

**Figure 2:**
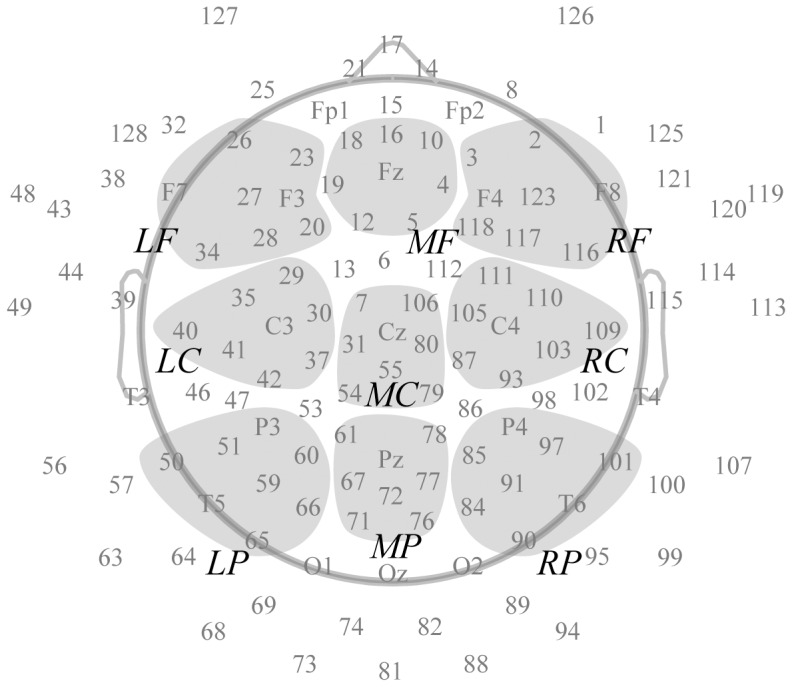
Schematic representation of a 128-channel montage and 9 clusters of electrodes for analysis. The 9 clusters are depicted in gray (LF, Left frontal; MF, Middle frontal; RF, Right frontal; LC, Left central; MC, Middle central; RC, Right central; LP, Left Parietal; Middle Parietal; RP, Right Parietal).

An electroencephalogram (EEG) was measured from 128 electrodes using the Geodesic sensor net (Geodesic EEG System 300, Electrical Geodesics Inc., USA), referenced online to vertex (Cz). All electrode impedances were reduced to less than 50 kΩ. The recorded data were sampled at 500 Hz. The continuous EEG data were digitally filtered (0.5–30 Hz elliptic IIR filter) after being re-referenced to an average reference using the EEGLAB toolbox (Delorme and Makeig, 2004). The continuous EEG was epoched into 1500-ms data (−700 to +800 ms from target onset) and baseline corrected (−200 to 0 ms from prime onset). The baseline correction might distort the ERP via possible transient signal differences in the baseline interval. However, in pre-stimulus period of our study, the subjects were required to push the response button, which activity was of little importance to our analysis. So we computed baseline-corrected ERP to stress statistical comparison between sensors and conditions. The trials containing artifacts exceeding ±70 mV in amplitude were rejected from further analysis. The mean number of trials after artifact rejection for low tendency group was 57.9 (SD = 11.5) in the negative prime condition, 57.1 (SD = 10.8) in the neutral prime and 58.0 (SD = 10.8) in the positive prime condition and the mean number of trials for high tendency group was 58.3 (SD = 17.8) in the negative prime condition, 56.6 (SD = 17.2) in the neutral prime and 60.8 (SD = 14.1) in the positive prime condition. Electrodes were classified into 9 regions: frontal, central, and parietal regions ×left, middle, and right regions. Each region consisted of 8 electrodes (Fig. 2). Mean amplitudes were computed at 3 time windows for each participant and type of prime; the first window was from the early component to the prime (P2 to primes, −300 to −200 ms). The second was from the early component to the target (P2 to targets, 200 to 300 ms). The third was from the late component to the target (LPP to targets, 500 to 800 ms). Then these amplitudes were averaged separately for each prime valence (negative, neutral, and positive) and misattribution tendency (high tendency, low tendency). A 3 prime valence (negative, neutral, positive)×2 misattribution tendency (high, low)×9 region (LF, MF, RF, LC, MC, RC, LP, MP, and RP) repeated measures ANOVA was performed for each component. The Greenhouse-Geisser **ε** correction was applied in order to adjust the degrees of freedom of the F ratios as necessary. All significant or marginally significant interaction effects were decomposed with simple main effects comparisons and post hoc analyses with Bonferroni corrections.

In addition, we performed a topographical analysis of the ERP data using Cartool software (http://brainmapping.unige.ch/Cartool.php). Segments of stable voltage topography were defined by using a topographic atomize and agglomerate hierarchical cluster analysis (T-AAHC) in the grand-averaged ERP's across conditions and groups over the post-prime EEG segment length of 1000 ms. This procedure was applied with several constraints: the maps had to remain stable for 20 ms and the maximum correlation between different topographies should not exceed 92%. The optimal number of these template maps was determined by the combination of a modified cross-validation and the Krzanowski–Lai criterion [20], [21]. In the next, a fitting procedure was conducted, which fits the dominant maps in the group-averaged to the ERPs of each individual participant. We conducted a repeated measures ANOVA on the map durations with the factors 3 prime valence (negative, neutral, positive)×2 misattribution tendency (high, low)×map configuration (number of microstates). The Greenhouse-Geisser **ε** correction was applied in order to adjust the degrees of freedom of the F ratios as necessary. All significant or marginally significant interaction effects were decomposed with simple main effects comparisons and post hoc analyses with Bonferroni corrections.

## Results

### Behavioral results

As a result of the classification based on the 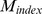, 11 participants were allocated in the high tendency group (the mean age was 23.7 (SD = 1.22), and 1 woman) and 9 participants in the low tendency group (the mean age was 25.4 (SD = 5.07), and 2 women). The mean 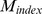 was 0.15 (SD = 0.11) for high tendency and −0.04 (SD = 0.03) for low tendency.

**Figure 3:**
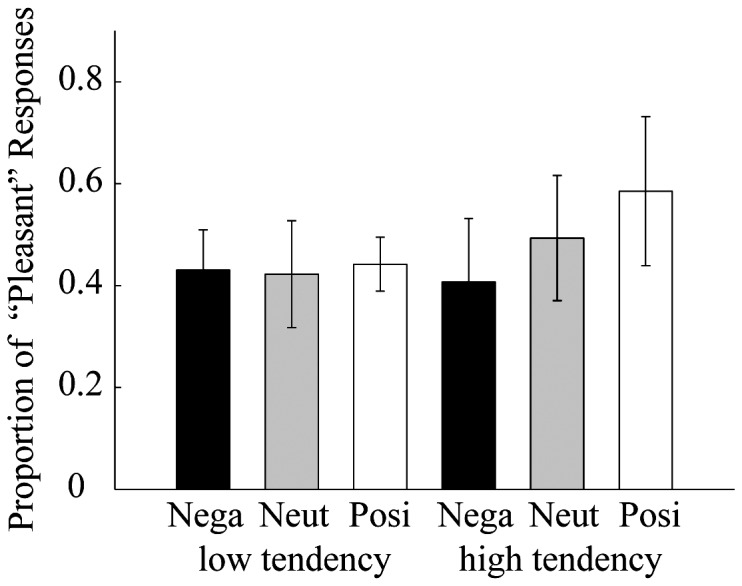
Proportion of “pleasant” responses as a function of prime valence and misattribution tendency. Error bars represent the standard deviation.

Figure 3 shows the proportion of “pleasant” responses as a function of prime valence and misattribution tendency. A 3 prime valence (negative, neutral, and positive)×2 misattribution tendency (high, low) repeated measures ANOVA was performed for the proportions of “pleasant” responses in each prime condition. The analysis of proportions shows a significant main effect of the prime valence [F (2, 36) = 16.52, p<.001]. Participants were most likely to judge targets as pleasant following positive primes compared to negative primes. However, there is no main effect of the misattribution tendency [F (1, 18) = 1.86, p = 0.19]. The interaction of prime valence and misattribution tendency reached significance [F (2, 36) = 12.82, p<.001]. The test for simple effects revealed that there was a significant effect of the prime valence for high tendency [F (2, 36) = 32.21, p<.001] and that it was not a significant effect for low tendency [F (2, 36) = 0.32, p = .682]. Post hoc comparisons showed that all 3 prime conditions for high tendency were significantly different from each other [all ts>4, ps<.001]. As to the reaction times, there is no significant main effect [prime valence: F (2, 36) = 0.41, p = 0.42, and misattribution tendency: F (1, 18) = 0.44, p = 0.38] and interaction [F(2, 36) = 0.89, p = 0.42 ]. These results indicate that the 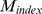 was suitable for the classification of AMP effects.

### ERP results to primes

**Figure 4:**
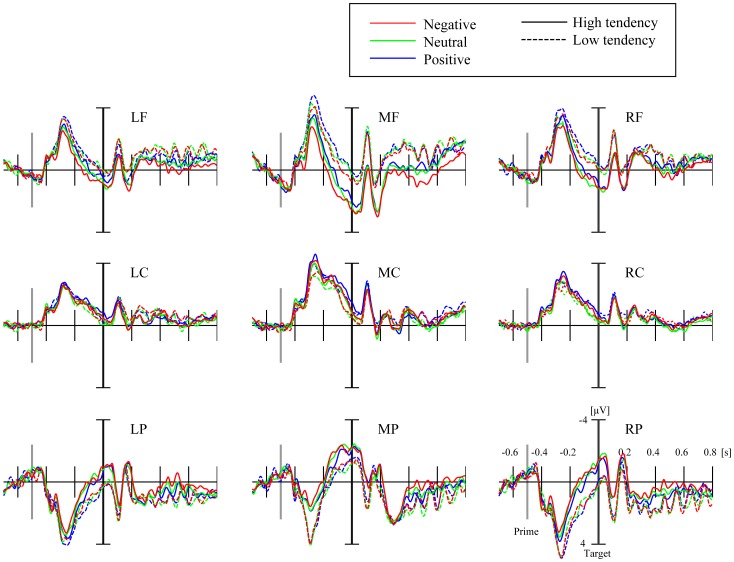
Grand-averaged ERPs in the 9 clusters for prime valence (negative, neutral, and positive) and misattribution tendency (high, low).

Figure 4 illustrates the grand-averaged ERPs to prime valence (negative, neutral, and positive) in the group of misattribution tendency (high, low) at each region. The ERPs were based on the target onset, and the prime onset was shown in the gray bar (−500 ms from target onset). Table 1 shows the mean amplitudes and standard deviations of ERPs for the negative, neutral, and positive primes for the groups of low and high misattribution tendency.

**Table 1:** Mean amplitudes and standard deviations of ERPs in 9 clusters for negative, neutral, and positive primes in two groups of high and low tendency.

Component	Cluster	Mean amplitudes and standard deviations											
		low tendency						high tendency					
		negative		neutral		positive		negative		neutral		positive	
		M	SD	M	SD	M	SD	M	SD	M	SD	M	SD
Prime P2													
	Left-frontal	−2.12	1.10	−1.81	0.75	−1.82	0.97	−1.53	0.92	−1.30	0.91	−0.96	0.83
	Middle-front	−3.09	1.37	−2.46	1.04	−2.36	1.21	−1.61	1.96	−1.27	2.08	−0.66	2.46
	Right-frontal	−2.79	0.77	−2.14	0.79	−2.34	0.87	−1.88	1.33	−1.50	1.09	−1.31	1.25
	Left-central	−2.00	1.04	−1.66	0.77	−1.87	1.01	−2.04	0.77	−1.81	0.77	−1.82	0.69
	Middle-centr	−3.24	1.04	−2.69	1.10	−2.96	1.14	−3.55	1.16	−3.13	1.31	−3.34	1.16
	Right-central	−2.29	1.41	−1.92	1.07	−2.11	1.14	−2.54	1.19	−2.15	1.02	−2.27	1.05
	Left-parietal	2.93	1.20	2.48	0.81	2.56	0.99	2.27	1.60	1.94	1.40	1.68	1.79
	Middle-pariet	1.34	1.20	1.21	1.15	1.08	1.24	0.27	1.14	0.01	1.07	−0.31	1.32
	Right-parietal	3.29	1.55	2.85	1.36	2.96	1.48	1.89	1.22	1.51	1.23	1.32	1.18
Target P2													
	Left-frontal	−0.77	1.17	−1.19	1.26	−1.18	1.42	−0.56	0.84	−0.59	0.77	−0.38	0.90
	Middle-front	−1.22	1.48	−1.26	1.07	−1.07	1.28	0.01	1.59	0.05	1.51	0.41	1.99
	Right-frontal	−1.28	1.13	−1.17	0.67	−1.16	0.97	−0.71	0.97	−0.60	0.58	−0.60	0.87
	Left-central	−0.70	0.79	−0.82	0.85	−0.95	1.02	−0.34	0.54	−0.42	0.43	−0.47	0.50
	Middle-centr	−0.55	0.69	−0.47	1.00	−0.55	0.59	−0.21	1.04	−0.28	0.87	−0.34	0.79
	Right-central	−0.73	1.14	−0.57	0.70	−0.62	0.79	−0.58	1.01	−0.54	0.87	−0.70	0.87
	Left-parietal	1.19	1.09	1.14	0.68	1.11	0.97	0.92	1.37	0.99	1.08	0.75	1.13
	Middle-pariet	1.89	1.16	2.09	1.19	1.95	1.20	1.67	1.24	1.53	0.99	1.49	1.25
	Right-parietal	1.59	1.09	1.70	0.89	1.70	0.99	1.07	1.21	0.87	1.04	0.71	1.13
Target LPP													
	Left-frontal	−0.68	0.50	−1.11	1.25	−1.10	0.79	−0.65	0.56	−0.53	0.53	−0.17	0.67
	Middle-front	−1.08	0.94	−1.52	0.92	−1.27	0.93	−1.02	1.11	−0.94	0.79	−0.20	0.91
	Right-frontal	−0.48	0.87	−0.75	0.80	−0.57	0.62	−0.53	0.69	−0.47	0.52	−0.18	0.46
	Left-central	−0.45	0.48	−0.30	0.52	−0.53	0.56	−0.30	0.34	−0.15	0.27	−0.21	0.32
	Middle-centr	−0.82	0.47	−0.37	0.48	−0.64	0.35	−0.48	0.50	−0.33	0.49	−0.49	0.43
	Right-central	−0.27	0.57	−0.17	0.46	−0.23	0.51	−0.30	0.45	−0.02	0.45	−0.23	0.49
	Left-parietal	0.86	0.82	1.13	0.51	0.89	0.45	0.73	0.46	0.63	0.41	0.28	0.41
	Middle-pariet	0.62	0.61	1.04	0.78	0.80	0.75	0.35	0.67	0.34	0.55	−0.05	0.52
	Right-parietal	0.80	0.54	1.20	0.63	1.17	0.81	0.74	0.70	0.63	0.57	0.29	0.73

The analysis of P2 to primes showed a significant main effect of prime valence [F (2, 36) = 8.60, p<.005]. The post hoc test indicated significant differences between negative and neutral (p<.01) and between negative and neutral stimuli (p<.05). The significant main effect of the region was also observed [F (8, 144) = 59.41, p<.001]. The interaction between the prime valence and the region reached significance [F (16, 288) = 9.49, p<.001]. The tests of simple effects indicated significant effects of the prime valence at all regions except the left central region [all Fs (2, 36)>4.12, all ps<.024]. Subsequent multiple comparisons revealed that smaller P2 to negative compared to both neutral and positive at the middle and right frontal and middle central regions, smaller P2 to negative compared to positive at the left frontal and smaller P2 to negative compared to neutral at the right central regions, while larger P2 to negative compared to both neutral and positive at the left and right posterior regions and larger P2 to negative compared to positive at the middle posterior region (all ps<.05). The interaction between the misattribution tendency and the region also reached significance [F (8, 144) = 3.34, p<.005]. The tests of simple effects indicated the significant effects of the misattribution tendency at the middle-parietal [F (1, 18) = 5.54, p<.05] and right-parietal regions [F (1, 18) = 6.21, p<.05]. At the middle- and right-parietal regions, P2 to primes was significantly larger for low tendency than for high tendency. However, there was no significant correlation between amplitude of P2 to primes and the misattribution index (Pearson's correlation coefficient, r = −0.27 p = 0.24 (the middle-parietal region), r = −0.37 p = 0.10 (the middle-parietal region)).

### ERP results to targets

The analysis of P2 to targets showed a significant main effect of the region [F (8, 144) = 19.70, p<.001], but no other effects and interaction involved the factors of either the prime valence or the misattribution tendency.

The analysis of LPP to targets showed a significant main effect of the region [F (8, 144) = 31.12, p<.001]. The interaction between the prime valence and the region reached significance [F (16, 288) = 2.17, p<.01]. The tests of simple effects indicated significant effects of the prime valence at the right central region [Fs (2, 36) = 3.89, p<.05]. Subsequent multiple comparisons revealed that smaller LPP to negative compared to positive at the right central region (p<0.05). The interaction between the misattribution tendency and the region also reached significance [F (8, 144) = 3.32, p<.005]. Further analyses revealed significant effects of the misattribution tendency at the left- [F (1, 18) = 4.74, p<.05] and middle-parietal regions [F (1, 18) = 5.94, p<.05], indicating that the LPP to targets was larger for low tendency than for high tendency. The 3-way interaction among prime valence, misattribution tendency, and region also reached significance [F (16, 288) = 2.72, p<.001]. The simple interaction effects of the prime valence and the misattribution tendency revealed a significant effect at the right-parietal region [F (2, 36) = 5.26, p<.05]. A further test at the right-parietal region revealed that the simple effect of the prime valence was significant for high tendency [F (2, 20) = 3.72, p<.05] but not significant for low tendency [F (2, 16) = 2.76, p = .077]. A post hoc analysis revealed that the LPP amplitude to negative prime was larger than the positive prime in high tendency. In addition, there was a significant correlation between amplitude of LPP to targets and the misattribution index (Pearson's correlation coefficient, r = −0.48 p<.05 (the middle-parietal region), r = −0.48 p<.05 (the left-parietal region)). Interestingly, there was also a significant positive correlation between amplitude of LPP to targets and P2 to primes in the middle-parietal region (Pearson's correlation coefficient, r = 0.55 p<.05).

### Topographic pattern analysis

**Figure 5:**
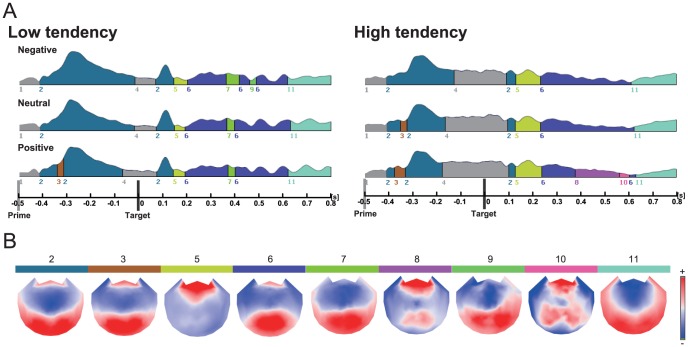
(A) Segments of stable map topography are shown group-wise (left: Low tendency, right: High tendency) for each stimulus condition. (B) Map topographies with the bar of corresponding color; positive voltages in red, negative in blue. These maps are 2-D projections of the 3-D electrode configuration (view from above, nasion on top).

Figure 5 shows the results of the topographical EP mapping of the grand-averaged data for each group and prime condition. In post prime period (−500 to 0 ms), for all conditions and groups, three maps (maps 1, 2, and 3) arose successively. The first map was common for both groups and for all stimuli. Map 2 peaked shortly 200 ms after the prime onset, which corresponds to the P2-like component. A fitting procedure was carried out on Maps 2 and 3 to test the differences at a statistical level (temporal interval: −416 to 0 ms). The durations of these maps were then extracted and compared by means of a repeated measure ANOVA with the factors 3 prime valence (negative, neutral, positive)×2 misattribution tendency (high, low)×map configuration (map 2 and 3) (We excluded the duration of Map 4 from furtherthe analysis because the map bestrode the onset of the target). The analysis of showed a significant main effect of valence [F(2, 36) = 8.22, p<.005, longer duration for positive than for negative stimuli (p<.01)] and map [F (1, 18) = 13.1, p<.005, longer duration for Map 2], and a significant interaction between the valence and map [F(2, 36) = 3.71, p<.05]. Subsequent multiple comparisons revealed that shorter duration to negative compared to positive only for Map 2 (p<.05). The interaction between the misattribution tendency and the map did not reach significance [F (1, 18) = 1.17, p = 0.29]. A repeated measure ANOVA with the factors 3 prime valence ×2 misattribution tendency for Map 2 showed a marginally significant effect of the misattribution tendency [F (1, 18) = 56.8, p = 0.88], which revealed that low tendency group exhibited the longer duration for map 2 than high tendency group.

For the period of 0 to 800 ms after target onset, eight maps (maps 2, 5, 6, 7, 8, 9, 10 and 11) arose successively. We computed a repeated measure ANOVA with the factors 3 prime valence (negative, neutral, positive)×2 misattribution tendency (high, low)×map configuration (maps 2, 5, 6, 7, 8, 9, 10 and 11). The analysis of showed a significant main effect of map [F(7, 126) = 4.31, p<.005]. Importantly, the interaction between the misattribution tendency and the map reached significance [F (7, 126) = 3.31, p<.005]. The tests of simple effects indicated the significant effects of misattribution tendency on Map 5 [F (1, 18) = 7.21, p<.05, longer duration for high tendency group] and Map 7 [F (1, 18) = 6.39, p<.05, longer duration for low tendency group]. Map5 peaked around 200 ms after the target onset, which shared several electrophysiological properties with the early posterior negativity (EPN). Map 7 started shortly 350 ms after the target onset, which corresponds to the LPP-like component.

## Discussion

The aim of the present study was to identify the ERP correlates of the affect misattribution effect that emotional judgments of targets misattribute to the valence of primes. In line with previous studies [1], [6], our behavioral results showed that, overall, the valence of primes influenced participants' evaluations of targets. However, there were individual differences in the tendency toward a misattribution effect, and some participants did not show the effect. In our study, we divided the participants into two groups based on their index in expressing the tendency toward misattribution. We investigated how the ERP components were modulated by their tendencies to misattribution.

The analysis of P2 to primes showed a significant main effect of prime valence regardless of the misattribution tendency. P2 has been suggested to reflect early processing and to be involved in attention allocation. The effect of the prime valence in P2 suggests that emotional pictures interfere with early attention allocation, which is consistent with results from previous works [11], [13]. It is important to note that P2 to primes was significantly larger in low tendency than in high tendency at the middle- and right-parietal regions. These results suggest that individual differences in the processing of primes influence the tendencies of subsequent target evaluation. In view of the relationship between attention allocation and P2 amplitude, this result indicates that attention to primes was higher in participants having a low tendency toward misattribution than in those having a high tendency. In the previous research [6], the affect misattribution effect was observed when participants ignored the primes; however, this effect disappeared when participants explicitly evaluated the primes before the targets were presented. Our results of a larger P2 for primes with low tendency provide neurophysiological support for the view that misattribution takes place when participants are unable to monitor and control the influence of their attitudes toward the prime on their judgments.

The effect of the prime valence did not appear in P2 to targets, but in LPP to target. Previous studies have suggested that the P2 index relatively automatically increases in selective attention, whereas LPP may be associated with evaluative processes following the presentation of emotional stimuli [11], [14], [15]. The result that P2 to targets was not significantly affected by the prime valence reflects the fact that early processing for neutral pictographs presented as targets was not affected by the prime valence. Additionally, no significant difference was observed between the high and low misattribution tendency groups, which suggests that attention to the targets was no different between the two groups.

As for the LPP component, the LPP to targets was larger for low tendency than for high tendency at the left- and middle-parietal regions. In addition, there was a significant correlation between amplitude of LPP to targets and the misattribution index. Ferrari et al., (2008) [22] showed that the LPP is clearly affected by attention during the categorization of natural scenes and directed attention enhanced the LPP both to emotional and neutral stimuli. Many other studies suggested that selective attention processing was reflected in the enhanced LPP amplitudes [23]–[26]. These results suggested that the less the misattribution index were, the more attention was allocated to the target. In addition, there was a significant positive correlation between amplitude of LPP to targets and P2 to primes. Taken together, the mechanism of the affective misattribution was suggested to be closely related to the attention allocation processing.

The LPP amplitude to targets following a negative prime was larger than to the positive prime for the high-tendency group, but the effect of the prime valence was not significant for low tendency. Our finding of the effect of the prime valence in LPP to targets for high tendency suggests that participants were affected by primes even when they judged neutral targets. Previous studies suggest that LPP may be associated with the evaluative processes following the presentation of emotional stimuli [11], [14], [15]. In addition, this discrepancy of LPP to targets between the two groups might reflect the bottom-up and top-down accounts of LPP to emotion [22], [27]–[30] in that the top-down effects of primes are different. Because of the top-down effects, the AMP high-tendency group was apt to misattribute the prime valence to neutral targets—that is, to regard neutral targets as emotional ones.

The results of the topographical analysis showed that the duration of Map 2 corresponding to P2 component was longer for low tendency group than high tendency group, which is consistent with the results of ERP analysis. The topographical analysis for the post target-onset period showed that the duration of Map 7 was longer duration for low tendency group, which is also consistent with the results of LPP component. On the other hand, the direction of the modulation as to Map 5 corresponding to EPN was contrary to that of LPP component. The EPN component indexes natural selective attention [26], [31], [32], while the more elaborated processing is reflected in the LPP [33], [34]. This contrast supports the hypothesis that the AMP high-tendency group was unable to monitor and control the influence of their attitudes and apt to regard neutral targets as emotional ones.

Our study suggested that attention allocation play an important role in the mechanism of affect misattribution. Attention allocation can substantially vary from trial to trial during the course of the experiment. In this view, trial-wise analyses, where trials with and without affect misattribution are analyzed separately, would be valuable. However, in a trial-wise manner, it is difficult to balance the trials of each condition for AMP unlike affective priming procedure, because the response to the neutral target cannot be predicted. So we analyzed the data in a group-wise manner.

## Conclusions

The AMP effect is known as the phenomenon in which the prime valence affects the affective reaction to the subsequent target. When participants judged neutral targets amiss according to the primes, the effect was reflected in the late processing of targets (LPP). Our results of P2 to primes support the view that attention to the prime is related to a misattribution effect. In addition, the topographic pattern analysis revealed that EPN-like component to targets was correlated with the difference of AMP tendency as well as P2 to primes and LPP to targets. Taken together, the mechanism of the affective misattribution was closely related to the attention allocation processing. Our findings provide neural evidence that evaluations of neutral targets are misattributed to emotional primes.
